# Anterior Abdominal Wall Necrotizing Fasciitis Following Laparoscopic Sleeve Gastrectomy With Hiatal Hernia Repair: A Case Report

**DOI:** 10.7759/cureus.63470

**Published:** 2024-06-29

**Authors:** Hope Fozo, Arani Thirunavukarasu, Taiwo Ogundolie, Frederick Tiesenga

**Affiliations:** 1 Surgery, Suburban Surgery Center, Chicago, USA; 2 Clinical Sciences, St. George's University School of Medicine, St. George, GRD; 3 General Surgery, West Suburban Medical Center, Chicago, USA

**Keywords:** bariatric surgery complication, hiatal hernia repair, weight loss and obesity, bariatric surgery complications, obesity and necrotizing fasciitis, abdominal wall necrotizing fasciitis, surgical complication, laparoscopic sleeve gastrectomy complications

## Abstract

Necrotizing fasciitis (NF), commonly known as necrotizing soft tissue infection (NSTI), or flesh-eating disease is a rare but rapidly fatal aggressive bacterial infection of soft tissue and deep skin that results in the destruction of the underlying fascia. Symptoms include fever, tachycardia, hypotension, leukocytosis, pain, and large areas of red and swollen skin. Early diagnosis and aggressive management are compulsory for a better prognosis. In this case report, we present a 58-year-old obese woman who initially presented to the emergency department three weeks post-sleeve gastrectomy with hernia repair and was initially suspected of having a large, uncomplicated abdominal wall abscess. Several repeated drainages of the abdominal wall abscess and continued deterioration of the patient revealed foul-smelling, necrotic tissue and the subsequent diagnosis of NF. This case report highlights the importance of high clinical suspicion for NF and early, aggressive debridement and treatment to improve patient outcomes.

## Introduction

Necrotizing fasciitis (NF), or flesh-eating disease is a rare but commonly aggressive bacterial infection of the soft tissue and deep skin that results in the destruction of the underlying fascia. The causative agents are commonly toxin-producing, highly virulent organisms, and the disease is typically polymicrobial; however, the most commonly implicated organisms are gram-positive, especially Group A Streptococcus. NF can be rapidly fatal due to severe systemic toxicity and sepsis if not recognized and treated promptly; thus, early recognition is crucial [1]. Diabetes is the most important risk factor for the development of NF [2-8]. Other risk factors include obesity, alcoholism/liver disease, IV drug use, age > 60 years, peripheral vascular diseases, and an immunocompromised state. Triggers for NF include major trauma, recent surgery, and childbirth [4-9]. Common areas affected by NF are the lower extremities, pericardial areas, and external genitalia with less than 25% of patients affected in the anterior abdominal wall which is typically due to penetrating trauma [7].

Because of its cryptic presentation, NF is often misdiagnosed leading to delayed treatment. Only 15% of patients are diagnosed correctly at the time of presentation and this delay in diagnosis and treatment plays a huge role in the high mortality rate [4,10]. Some symptoms that should raise a clinician’s degree of suspicion include a patient with vital abnormalities such as tachycardia, hypotension, and physical exam findings of fever, erythema, induration, crepitation, swelling, and pain that may be out of proportion to exam findings [8]. Patients with the following risk and precipitating factors should make a physician warier of the diagnosis of NF. These include diabetes (greatest risk factor), obesity, IV drug use, recent surgery or trauma, childbirth, malignancy, immunocompromised state, liver disease/alcoholism, and age > 60 [4,11]. In this article, we present a case of NF whose initial presentation was confused with a complicated abdominal abscess.

## Case presentation

History and initial presentation

In recent years, laparoscopic sleeve gastrectomy (LSG) has gained traction as a viable weight loss surgery in morbidly obese patients. This technique has been favored over the previously used laparoscopic Roux-en-Y gastric bypass (RYGB) due to its technical simplicity, minimally invasive nature of the surgery, and eliminating the need for gastrointestinal anastomosis [12].

Here, we present a 58-year-old woman with a BMI of 49.61 who was evaluated for morbid obesity. A recommendation for an LSG was made, and the patient was cleared for surgery by a multidisciplinary team. The patient had no significant previous medical history or comorbidities and was not on any medication prior to the surgery. Her past surgical history and procedures consist of a cholecystectomy, a hysterectomy, a colonoscopy, and an esophagogastroduodenoscopy.

Once adequate anesthesia was provided, the patient was intubated and received appropriate IV antibiotics within one hour of skin incision. A LSG was performed and during the procedure, a moderate-sized hiatal hernia was discovered, and the decision was made to repair it. The operation went on without any complications and was completed within 1 hour and 24 minutes after which the patient was successfully extubated in the operating room. Her immediate post-op period was uncomplicated with moderate post-surgical pain. The patient was subsequently discharged on post-op day 1 with advice to follow up in the outpatient surgical center.

Three weeks following the procedure during her outpatient surgical clinic visit, the patient was referred to the emergency department (ED) for continuous abdominal pain and hypotension. The pain was progressively worsening and was made worse by movement. The patient also complained of abdominal redness and decreased appetite. Initial examination in the ED revealed the patient to be mildly hypotensive with blood pressure of 73/58 mm Hg but the patient was afebrile and not tachycardic. Physical examination of the abdomen demonstrated erythema over previous surgical sites, a 4 cm x 3 cm poorly defined area of erythema over the anterior abdominal wall with minimal induration and tenderness, and a 6 cm x 6 cm tender, indurated area over the left lower abdomen. Labs were ordered and despite no fluctuance felt, a computed tomography (CT) scan was ordered due to concerns for possible abscess, and broad-spectrum IV antibiotics with Piperacillin/Tazobactam were administered while awaiting CT results.

Investigations

**Figure 1:**
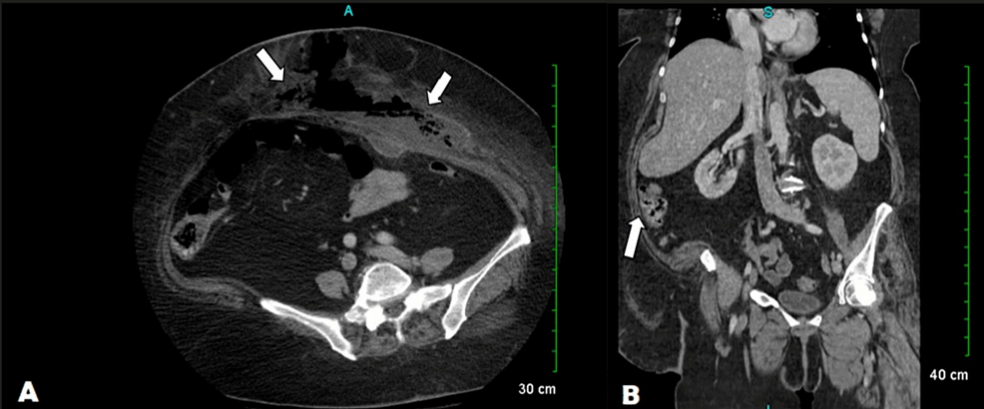
Air fluid collection present in the subcutaneous fat of the anterior lower abdominal wall measuring approximately 17 × 4 cm (A) with a fluid collection abutting the anterior abdominal wall measuring 3.4 × 1.3 cm (B)

ED labs for our patient revealed leukocytosis (WBC: 28.7), acute kidney injury (AKI) with a BUN of 36 and creatinine of 1.36, and hypokalemia (Table 1). CT revealed an air-fluid collection in the lower anterior abdomen measuring 17 x 4 cm (Figure 1A). There was also a small fluid collection abutting the anterior abdominal wall measuring 3.4 x 1.3 cm (Figure 1B), findings suggestive of an abdominal wall abscess with cellulitis.

**Table 1:** Patient lab values at initial presentation to the emergency department prior to surgical consultation MCV: mean corpuscular volume; MCH: mean corpuscular hemoglobin; MCHC: mean corpuscular hemoglobin concentration; RDW: red cell distribution width; MPV: mean platelet volume; BUN: blood urea nitrogen

Component	Patient values	Reference range and values
WBC	28.7	4.0-11.0 k/mm cu
Platelets	246	140-450 k/mm cu
Hemoglobin	11.9	12.0-15 g/dL
Hematocrit	36.1	34.7-45.1%
MCV	90.6	80.0-100.0 fL
MCH	29.8	26.0-34.0 pg
MCHC	32.9	32.5-35.8%
RDW	14.5	11.9-15.9%
MPV	8.2	6.8-10.2 fL
Glucose	241	70-99 mg/dL
Calcium	7.7	8.6-10.3 mg/dL
Sodium	135	135-145 mmol/L
Potassium	3.1	3.5-5.1 mEq/L
CO_^2^_	24	21-31 mEq/L
Chloride	99	98-109 mEq/L
BUN	36	7-25 mg/dL
Creatinine	1.36	0.6-1.30 mg/dL

Management and postoperative care

The surgery team was consulted, and the patient was transported to the operating room for incision and drainage of the abdominal wall abscess. In the operating room, incisions were made over four previous port sites and dissection was carried down until the abscess cavity was encountered. About 600 mL of pus was drained from the abdomen and culture samples were obtained which grew mixed skin flora. The patient was transported to the post-anesthesia care unit (PACU) in stable condition and continued IV fluids, IV Piperacillin/Tazobactam 4.5 g every eight hours, and pain medications.

On day 3 post-admission and day 1 post-exploration, the patient had continued cellulitis and leukocytosis. Repeat surgical exploration was recommended due to concerns of persistent underlying abscess. The patient was taken to the operating room where the abdomen was bluntly explored. Small residual pockets of abscesses were found, opened, and rinsed out meticulously. The wounds were packed open with moistened Kerlix.

On day 4 post-admission, day 1 post-second exploration, washout, and drainage of the abdominal wall abscess, a new area of fluctuance was noted and it was recommended that the patient return to the operating room for further exploration. In the operating room, the two left quadrant incisions were combined using cautery and it was at that time that necrotic and foul-smelling tissue was noted at the level of the fascia between the two incisions consistent with NF. At that time, a total area of approximately 30 × 10 cm of necrotic tissue was removed including excision of the previous incision, and drainage sites were thoroughly debrided to viable tissue with the placement of Penrose drains. The wound was packed and left open, and the patient was continued on IV fluids, pain management, and continued on Piperacillin/Tazobactam. At this time, the patient was empirically started on Clindamycin and Vancomycin for broader coverage.

**Figure 2:**
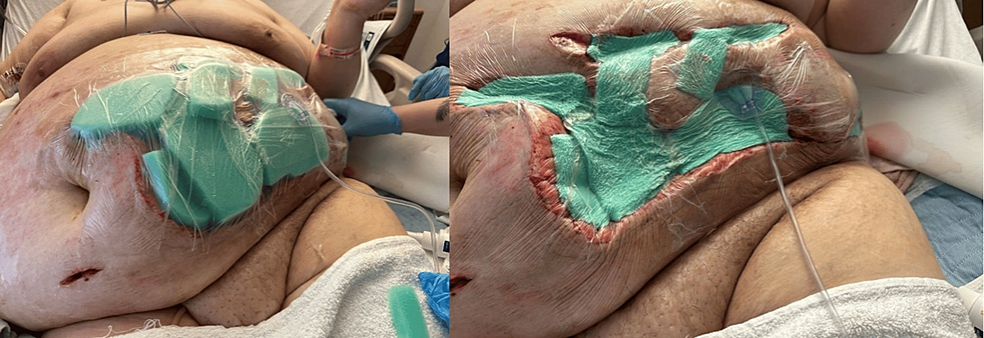
Negative wound vacuum attached to anterior abdominal wound following surgical debridement

**Figure 3:**
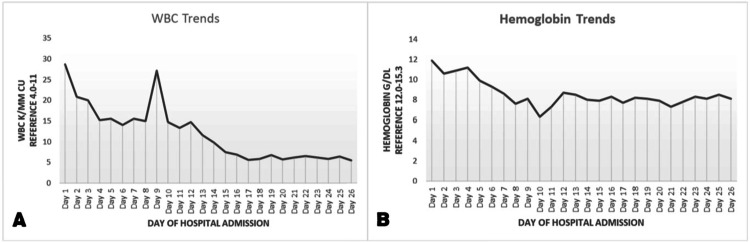
Patient's WBC trends (A) and hemoglobin trends (B) during entire hospital stay

On day 5 post-admission, day 2 post-second exploration, and day 1 post-abdominal wall debridement, the patient was taken back to the operating room for further debridement with the placement of a negative pressure wound therapy (Figure 2). Later that evening, the wound vacuum was noted to be full of bloody drainage and the patient continued to experience leukocytosis (Figure 3A) with continuous declining hemoglobin (Figure 3B).

The following day, additional necrotic tissue was noted, and the patient was taken back to the operating room for her third debridement. The patient subsequently continued daily debridement of necrotic tissue; however, after her fifth debridement, the patient experienced increased work of breathing during extubating and was immediately reintubated, transferred to the intensive care unit (ICU), and sedated with propofol. Daily debridement was canceled due to acute hypoxic respiratory failure.

**Figure 4:**
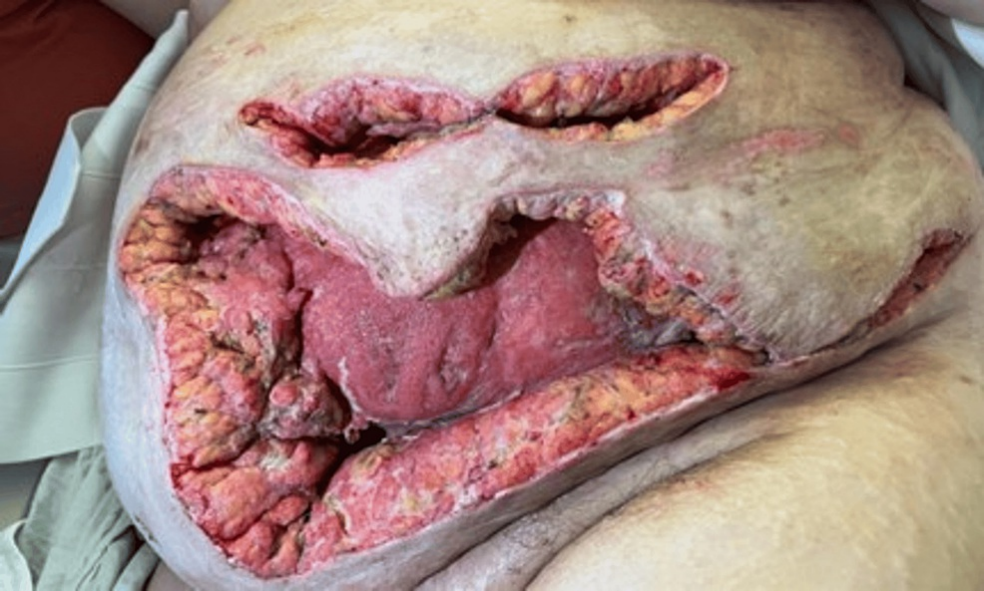
Anterior abdominal wall showing extensive necrotic tissue

On day 10 post-admission, multiple areas of continued infection were found in the left upper and lower quadrants (Figure 4). The patient was subsequently transported to the operating room where approximately 40 cm^2^ of necrotic tissue was debrided. Because of continuous infection, it was decided not to place a wound vacuum at this time. Following completion of the procedure, the patient was transferred back to the ICU still intubated and in stable condition. After seven days in the ICU, anemia necessitated a blood transfusion, undergoing several more debridements, and a single episode of atrial fibrillation, our patient was finally in stable condition with resolved NF. She was discharged to a skilled bariatric care center to undergo the grueling journey of healing and recovery.

## Discussion

NF is typically diagnosed based on high clinical suspicion due to history and clinical examination but can only be confirmed with surgery [1,3]. This indicates that an accurate diagnosis of NF is based on clinical judgment by the clinicians; however, since NF is a rare disease, most clinicians are unaware of its initial presenting signs and symptoms thus delaying treatment [2,4]. Therefore, regardless of the low incidence of NF, it is imperative that clinicians are aware of the disease and its potential dangers, especially in the presence of risk and/or precipitating factors, to ensure the timely administration of appropriate treatment [2,13]. There is a proposed scoring system known as the laboratory risk indicator for necrotizing fasciitis (LRINEC) which is utilized by clinicians to determine the probability of a patient having NF versus another skin infection such as cellulitis. The scoring system is based on six variables that add up to a total of 14 points and is shown in Table 2. The six variables are as follows: C-reactive protein, WBC count, hemoglobin levels, sodium levels, creatinine levels, and glucose levels [2-4,9,11]. The score ranges from 0 to 14. A score of ≤5 indicates minimal risk (<50% probability) of having NF. A score of ≥8 indicates a high risk (>75% probability) of NF being present. As the C-reactive protein value was not part of the lab results analyzed at the initial presentation, the LINREC score for our patient could not be determined.

**Table 2:** The laboratory risk indicator for necrotizing fasciitis

Parameter	Value range	Score
Hemoglobin (g/dL)	>13.5	0
	11-13.5	1
	<11	2
WBC (10^9/L)	<15	0
	15-25	1
	>25	2
Sodium (mmol/L)	<135	2
Creatinine (μmol/L)	>141	2
Glucose	>180	1
C-reactive protein	>150	4

The clinical manifestations of NF vary widely as it lacks the obvious early cutaneous manifestations of infectious skin disease; however, NF is usually considered in patients who present with signs of skin infection following recent surgery [1,3,8-12]. Symptoms that may be present earlier in the disease include disproportionate pain. As the disease progressively worsens, patients present with symptoms of fever and possibly sepsis [1,4]. Common clinical findings associated with NF are patchy discoloration of the skin, extreme pain, which may be disproportionate to the physical exam findings, undefined swelling, fast progression to severe edema, necrosis and crepitus, and possible compartment syndrome due to increasing intra-compartmental pressure [5-8]. Patients with underlying conditions such as diabetic neuropathy could experience minimal pain making the diagnosis of NF more complicated in this population [1,10].

Additionally, a few common postoperative findings include subcutaneous emphysema, alterations in fat density and small, non-encapsulated crescent-shaped fluid collections, pneumoperitoneum, and postoperative ileus [1]. It is important to recognize the chronology of these findings to ensure they are not confused with any complications such as NF [1]. One key method to confirm whether a patient has symptoms of normal postoperative findings or a complication of surgery is through imaging [1]. Imaging is relied upon where signs are unclear to aid in identifying the extent of the disease as complications of the infection [1]. Plain radiography may show increased soft-tissue thickness and opacity with the presence of subcutaneous air distinguishing NF from opaque foreign bodies such as surgical instruments that may have been left behind [1].

**Figure 5:**
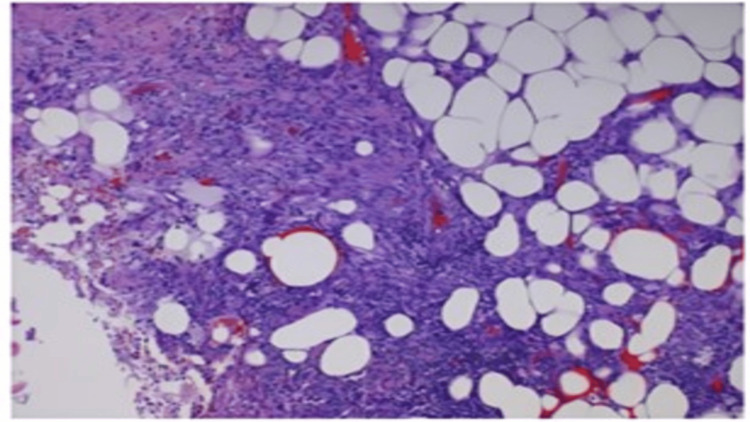
Histological segment of our patient’s tissue specimen demonstrating characteristic findings of necrotizing fasciitis including skin and subcutaneous tissue with abscess, fat necrosis, acute and chronic inflammation, and reactive changes

Histological criteria for diagnosing NF include necrosis of the deep fascia, polymorphonuclear neutrophils (PMNs) infiltration of the dermis and fascia, fibrin thrombi with necrosis of arterial and venous walls within the fascia, and the presence of microorganisms within the destroyed fascia and dermis [1] as observed in our patient’s tissue samples (Figure 5).

NF is often misdiagnosed as cellulitis or deep vein thrombosis which is why ultrasound is most used to rule them out [1,2]. Possible ultrasound findings that would indicate NF are turbid fluid collections near the deep fascia, thickening of the deep fascia, muscle swelling (features of myositis), and swelling of subcutaneous tissue (features of cellulitis) [1]. CT is the most sensitive imaging technique to detect any soft tissue air collections as demonstrated in our patient (Figure 1) and typically shows edema, stranding of subcutaneous tissue fat, asymmetrical thickening, fluid collections, and thickened regional muscles allowing rapid detection of NF [1,8]. However, MRI is the recommended imaging technique which shows hyper-intense subcutaneous tissue, deep fascial thickening, fluid collections in the deep fascia, and a hyperintense T2 signal [1].

**Figure 6:**
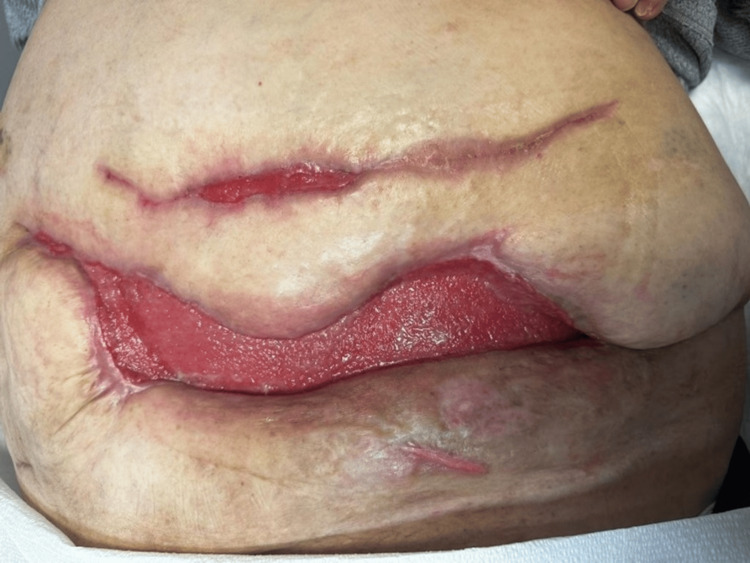
Healthy granulation tissue shown in our patient seven weeks post-last surgical debridement for necrotizing fasciitis

The prognosis of NF is directly correlated to the timing of surgical intervention indicating that early diagnosis and treatment results in the best prognosis [1]. It has been noted that some patients can heal without additional interventions; however, certain patients require more advanced treatment options. One study showed that of the three patients who suffered from NF of varying degrees, two of them required a skin graft to fully heal [8]. Typically, patients with a larger area affected by NF require a second reconstruction using abdominoplasty techniques once granulation tissue has formed which consists of a skin graft or a biological mesh [8]. Tissue grafting was discussed with our patient to speed up recovery; however, our patient decided to let her wound heal without grafting and continuous wound care (Figure 6). In spite of surgical intervention and treatment, NF carries a significantly high mortality rate [4]. It is expected that the mortality rate associated with NF can reach anywhere between 25-46% [4]. Regardless of the ongoing advances in medicine, the mortality rate for NF has not changed in recent decades [6]. Those with diminished immunity, AIDS, a history of radiotherapy or chemotherapy for malignant tumors, and any other co-morbidities are more susceptible to NF resulting in a worse prognosis [1,11].

## Conclusions

NF is a rare, but potentially fatal disease that requires a high degree of clinical suspicion to improve diagnosis time and prognosis due to its cryptic presentation. Despite our advancements in medicine and having a better understanding of this disease process, mortality remains high. As such, clinicians must maintain a high level of vigilance, acquire a comprehensive patient history, and perform a thorough physical exam to avoid overlooking critical information that may delay diagnosis and prognosis. Prompt diagnosis and effective debridement are crucial for lowering mortality rates.
